# Coherent and Noncoherent Joint Processing of Sonar for Detection of Small Targets in Shallow Water

**DOI:** 10.3390/s18041154

**Published:** 2018-04-10

**Authors:** Xiang Pan, Jingning Jiang, Si Li, Zhenping Ding, Chen Pan, Xianyi Gong

**Affiliations:** 1 College of Information Science and Electronic Engineering, Zhejiang University, Hangzhou 310027, China; jakejiangjn@zju.edu.cn (J.J.); janelisi@163.com (S.L.); dzpseu@139.com (Z.D.); gongxy@zju.edu.cn (X.G.); 2 Hangzhou Xuejun High School, Hangzhou 310012, China; Evepanchen@163.com

**Keywords:** spatial diversity, coherent processing, detection, sonar, at-lake experiment

## Abstract

A coherent-noncoherent joint processing framework is proposed for active sonar to combine diversity gain and beamforming gain for detection of a small target in shallow water environments. Sonar utilizes widely-spaced arrays to sense environments and illuminate a target of interest from multiple angles. Meanwhile, it exploits spatial diversity for time-reversal focusing to suppress reverberation, mainly strong bottom reverberation. For enhancement of robustness of time-reversal focusing, an adaptive iterative strategy is utilized in the processing framework. A probing signal is firstly transmitted and echoes of a likely target are utilized as steering vectors for the second transmission. With spatial diversity, target bearing and range are estimated using a broadband signal model. Numerical simulations show that the novel sonar outperforms the traditional phased-array sonar due to benefits of spatial diversity. The effectiveness of the proposed framework has been validated by localization of a small target in at-lake experiments.

## 1. Introduction

Signal fading due to target scintillation can cause large degradations in detection and estimation performance of the sonar system. Thus, spatial diversity is utilized in multistatic sonar for overcoming target scintillation by widely-spaced transmitters transmitting multiple signals from several angles to illuminate the ideally decorrelated aspects of a target [1,2]. In [1], an iterative minimization approach is proposed to exploit spatial diversity for mutual interference suppression and target range-Doppler imaging. Improvement of target detection is achieved through exploiting advantages of continuous illumination and spatial diversity [2]. Moreover, tracking accuracy can be further improved by exploiting spatial diversity [3,4]. In [3], due to the use of the aspect-dependent target diversity and Doppler features, the dim targets can be effectively tracked in high clutter environments. The aspect-dependent amplitude information is combined with range, bearing and Doppler measurements for correct estimation of number of targets and the better tracking results [4]. In addition, diversity gain can be enjoyed by multiple-input-multiple-output (MIMO) underwater acoustic communication systems for improving channel capacity [5,6,7].

As the special case of multistatic sonar, MIMO sonar enjoys the fact that the average signal-to-noise ratio (SNR) of the received signal is more or less constant due to spatial diversity gain [8,9,10,11]. In detection of a small target, MIMO sonar has a better resolution than synthetic aperture sonar (SAS) [9]. With combination of high-resolution beamforming and waveform diversity, MIMO sonar can achieve improvement of resolution [10]. The experimental results have shown that the MIMO sonar system has 12–15 dB additional diversity gain over its phased-array counterpart [11]. Spatial diversity includes the horizontal and vertical diversity. In underwater acoustic waveguides, vertical diversity attracts a lot of interest due to exploiting spatial diversity for dealing with multipath propagation resulting in time-delay spread. Diversity gain in a vertical direction is a function of array aperture rather than number of array elements. To maximize diversity gain, and minimize array aperture and computational complexity, the elements should be spaced at the order of the signal coherence length [12,13,14].

In this paper, we focus on coherent and non-coherent joint processing to achieve beamforming array gain and spatial diversity gain for improvement of detection performance of the traditional phased-array sonar. Due to building on the plane wave propagation model, phased-array sonar rarely utilizes vertical diversity for target detection. For considering practical applications, we utilize a horizontal array to receive target echoes. Meanwhile, several vertical transmitting arrays are widely-spaced to illuminate the likely target from different angles. The vertical diversity is exploited by the time-reversal (TR) mirror [15,16,17,18,19,20,21,22,23] to enhance target echoes and suppress reverberation by spatial-temporal focusing. For improving robustness of time-reversal focusing, an adaptive iterative strategy is considered in the processing framework. A probing signal (PS) is firstly transmitted, and then the received echo of a likely target is viewed as a guiding source for the second transmission. For simplicity, the novel sonar system is referred to as the distributed TR-MIMO sonar system. Due to the use of the horizontal diversity in phased-array sonar, the traditional phased-array sonar in this paper is also referred to as the phased-MIMO sonar. A similar system has been proposed in [24] where the pseudo time reversal is utilized to increase SNR. In addition, the effectiveness of our system has been validated by target localization in the at-lake experiments while the performance of the latter is evaluated by a lab tank experiment.

The rest of the paper is organized as follows. A coherent and non-coherent joint processing framework is proposed for target detection in Section 2. Section 3 evaluates diversity gain in improvement of detection performance by numerical simulations. The effectiveness of the joint processing framework is demonstrated by target localization in at-lake experiments in Section 4. Some conclusions are given in Section 5.

## 2. Coherent-Noncoherent Processing Framework

### 2.1. Transmitting Diversity

**Figure 1:**
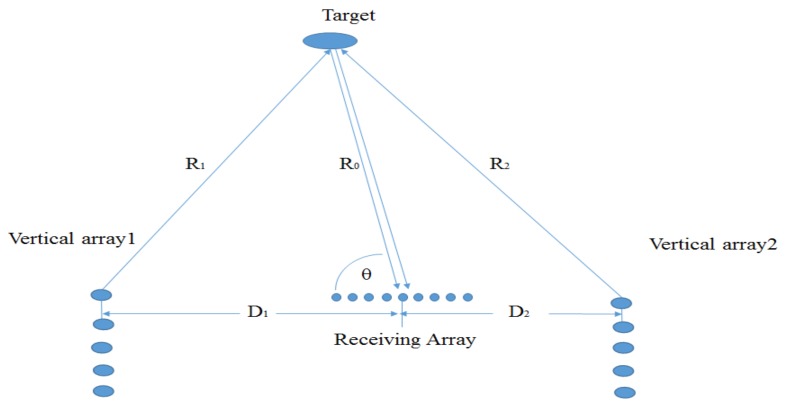
The array configurations for exploiting spatial diversity.

We consider a sonar system consisting of *M* vertical transmitting arrays that are widely-spaced to illuminate a target from multiple angles. Moreover, each vertical array has the element-spacing of eight wavelengths at 6 kHz to exploit the vertical diversity for time-reversal focusing. In [12,13,14], the element spacing of two wavelengths at 1.2 kHz is utilized to achieve spatial diversity. In [6], the element spacing is 2.25 wavelengths at 18 kHz. For simplicity, we assume that two neighbour vertical arrays are located at two ends of the receiving array as shown in Figure 1. The distance between two neighbour vertical arrays is required to meet the constraint condition [8,25]:

(1) $$\begin{array}{c} D_{1}+D_{2}\geq \frac{\lambda R_{0}}{\Delta x}, \end{array}$$

where Δx denotes target length, $D_{1}+D_{2}$ is the distance between two vertical arrays, $R_{0}$ denotes distance from the target to the receiving array, λ is wavelength, $\lambda =max\{\lambda_{1},\lambda_{2}\}$, and $\lambda_{1}$ and $\lambda_{2}$ respectively denote wavelength of two signals transmitted by two vertical arrays. Equation (1) means that two neighbour vertical arrays are not within the same receiving beamwidth of the target in the far field when the extended target is viewed as a receiving array with aperture Δx. In other words, two neighbour vertical arrays can see the different aspects of the target.

For exploiting spatial diversity for time-reversal focusing, a probe signal is firstly transmitted by an ominidirectional transmitter. Then, echoes of a likely target are received and transmitted back in a time-reversed order by the vertical arrays. Thus, the different aspects of the target are illuminated from different angles. Assume that the probing signal $S_{i}\left(f_{l}\right)$ {i=1,…,M,l=0,1,…,L−1} in the frequency domain is defined by

(2) $$\begin{array}{c} |S_{i}\left(f_{l}\right){|}^{2}=1/L,\\ \sum_{l=0}^{L-1}S_{i}\left(f_{l}\right)S_{j}^{*}\left(f_{l}\right)=\delta \left(i-j\right). \end{array}$$

Thus, the echo of the likely target received by the *m*th (m=1,2,…,M) vertical array is expressed as a vector

(3) $$\begin{array}{cc} X_{m}\left(f_{l}\right)= & H_{m}\lambda_{m}\left(f_{l}\right)G\left(f_{l}\right)S_{m}\left(f_{l}\right)\\ = & H_{m}{\tilde{S}}_{m}\left(f_{l}\right), \end{array}$$

where $G(f_{l})$ denotes the channel response from PS to the target, $H_{m}\left(f_{l}\right)$ denotes the channel response from the target to the *m*th vertical array, and $\lambda_{m}\left(f_{l}\right)$ denotes the target scattering coefficient. Let ${\tilde{S}}_{m}\left(f_{l}\right)=\lambda_{m}\left(f_{l}\right)G\left(f_{l}\right)S_{m}\left(f_{l}\right),$ which can be viewed as a second source for guiding time-reversal transmission. For simplicity, the term $\lambda_{m}\left(f_{l}\right)$ may be absorbed in $G(f_{l})$. In addition, the channel response $H_{m}\left(f_{l}\right)$ or $G(f_{l})$ can also be achieved by running the Kraken model [26] with a virtual source and the geoacoustic parameters [27,28].

The received echoes are time-reversed as:

(4) $${X^{*}}_{m}\left(f_{l}\right)=H_{m}^{*}\left(f_{l}\right)G^{*}\left(f_{l}\right)S_{m}^{*}\left(f_{l}\right)$$

after energy normalization, and the transmitted time-reversal signal can be expressed as

(5) $${\tilde{X}}_{m}\left(f_{l}\right)=\frac{{X^{*}}_{m}\left(f_{l}\right)}{\left|\right|{X^{*}}_{m}\left(f_{l}\right)\left|\right|.}$$

Thus, the time-reversal signal arriving at the target can be expressed as

(6) $$\begin{array}{c} \begin{array}{cc} Z_{m}\left(f_{l}\right)= & \sqrt{\frac{E}{M}}\frac{H_{m}^{T}\left(f_{l}\right)}{\left|\right|H_{m}\left(f_{l}\right)\left|\right|}\frac{H_{m}^{*}\left(f_{l}\right)}{\left|\right|H_{m}^{*}\left(f_{l}\right)\left|\right|}\frac{G^{*}\left(f_{l}\right)}{\left|\right|G^{*}\left(f_{l}\right)\left|\right|}S_{m}^{*}\left(f_{l}\right)\\ = & \sqrt{\frac{E}{M}}\beta_{m}\left(f_{l}\right)\frac{G^{*}\left(f_{l}\right)}{\left||G(f_{l})|\right|}S_{m}^{*}\left(f_{l}\right), \end{array} \end{array}$$

where $\beta_{m}\left(f_{l}\right)=|H_{m}\left(f_{l}\right){|}^{2}/\left|\right|H_{m}\left(f_{l}\right){\left|\right|}^{2}$, and the term E/M denotes that the total energy *E* is distributed over *M* vertical arrays. From Equation (6), we can see that the time-reversed signal has focused on the target due to the term $G\left(f_{l}\right)S_{m}\left(f_{l}\right)$ denoting the target echo.

From the beamforming perspective, time-reversal focusing can be regarded as active matched field processing (MFP) with the known environmental parameters. Hence, the spatial resolution of time-reversal focusing is equivalent to that of MFP in a waveguide. The range resolution ΔR is [29,30]:

(7) $$\Delta R=2\pi /(k_{1}-k_{2}),$$

where $k_{1}$ and $k_{2}$ are the horizontal wave numbers corresponding to the first and last effective modes, respectively. The depth resolution Δz is [30]:

(8) Δz=Z/L,

where *Z* is water depth, and *L* is the number of effective modes.

The maximum number of the excited modes in a waveguide can be estimated by [31]

(9) $$L=\frac{2Z}{\lambda }\sqrt{1-\frac{c^{2}}{c_{1}^{2}}}+\frac{1}{2},$$

where *c* is sound speed in water just above the seafloor, λ denotes wavelength, and $c_{1}$ is sound speed in the sediment just below the seafloor.

### 2.2. Broadband Signal Model

We further assume that the receiving array is composed of *N* elements with the inter-element spacing of half wavelength. Assume that target bearing is θ. The received echoes corresponding to the *m*th transmitted signal can be expressed as

(10) $$\begin{array}{c} \begin{array}{cc} r(f_{l},\tau_{m},\theta )= & \sqrt{\frac{E}{M}}a(f_{l},\theta )\zeta_{m}\left(f_{l}\right)\beta_{m}\left(f_{l}\right)\frac{G^{*}\left(f_{l}\right)}{\left||G(f_{l})|\right|}S_{m}^{*}\left(f_{l}\right)e^{-j2\pi f_{l}\tau_{m}}\\ & +n_{m}\left(f_{l}\right), \end{array}\; \end{array}$$

where $a(f_{l},\theta )$ denotes the array response vector, $\zeta_{m}\left(f_{l}\right)$ denotes the scattering coefficient, $\tau_{m}$ denotes the propagation time from the *m*th vertical array to the target and reflection back to the receiving array, and $n_{m}\left(f_{l}\right)$ denotes interference containing noise and reverberation.

### 2.3. Estimation of Target Bearing and Range

Target bearing can be estimated by [32]

(11) $$\hat{\theta }=\underset{\theta }{argmax}\sum_{m=1}^{M}{\left|\sum_{l=0}^{L-1}\frac{{a(f_{l},\theta )}^{H}}{\sqrt{N}}r\left(f_{l},\tau_{m},\theta \right)\right|}^{2}.$$

Equation (11) describes incoherent summation of multiple target echoes when the target is simultaneously illuminated by *M* orthogonal signals.

With the estimated $\hat{\theta }$, the beamformer output corresponding to the *m*th transmitted waveform is given by

(12) $$\tilde{y}\left(f_{l},\tau_{m}\right)=\frac{{a(f_{l},\hat{\theta })}^{H}}{\sqrt{N}}r_{m}\left(f_{l},\tau_{m},\hat{\theta }\right).$$

Correspondingly, the propagation time vector $\hat{\tau }$ can be estimated by [32]

(13) $$\hat{\tau }=\underset{\tau }{argmax}\sum_{m=1}^{M}{\left|\sum_{l=0}^{L-1}S_{m}\left(f_{l}\right)e^{j2\pi f_{l}\tau_{m}}\tilde{y}\left(f_{l},\tau_{m}\right)\right|}^{2},$$

where $\tau =[\tau_{1},\cdots ,\tau_{M}]$. Equation (13) denotes incoherent summation of outputs of matched filters.

Due to multipath propagation resulting in time-delay spread, we utilize the replica correlation integration processer [33] to replace the matched filter for estimating the target range in shallow water environments. Correspondingly, Equation (13) is rewritten as follows:

(14) $$\hat{\tau }=\underset{\tau }{argmax}\sum_{m=1}^{M}\sum_{u=0}^{U-1}\sqrt{\frac{2}{L}}{\left|\sum_{l=0}^{L-1}S_{m}\left(f_{l}\right)e^{j2\pi f_{l}\left(\tau_{m}+uT_{s}\right)}\tilde{y}\left(f_{l},\tau_{m}\right)\right|}^{2},$$

where $U=f_{s}T_{r}$, $f_{s}=1/T_{s}$ is the sampling rate, and $T_{r}$ denotes the signal time-spread length.

With the estimates of target bearing $\hat{\theta }$ and time-delay $\hat{\tau }$, we can calculate the target range $\hat{R}$ according to Figure 1:

(15) $$\hat{R}=\frac{1}{M}\sum_{m=1}^{M}\frac{D_{m}^{2}-c^{2}{\hat{\tau }}_{m}^{2}}{2(D_{m}cos\left(\hat{\theta }\right)-c{\hat{\tau }}_{m})},$$

where $D_{m}$ denotes distance from the *m*th vertical array to the center of the receiving array.

### 2.4. Target Detection

Furthermore, we evaluate detection performance of the sonar system with spatial diversity gain. For simplicity, assume that θ and τ are known in advance, and the detection problem can be cast as

(16) $$\left\{\begin{array}{c} H_{0}:\;\mathrm{Target}\;\mathrm{does}\;\mathrm{not}\;\mathrm{exist}\;\mathrm{at}\;\mathrm{bearing}\;\theta \;\mathrm{and}\;\mathrm{time}\;\tau ,\\ H_{1}:\;\mathrm{Target}\;\mathrm{exists}\;\mathrm{at}\;\mathrm{bearing}\;\theta \;\mathrm{and}\;\mathrm{time}\;\tau \;. \end{array}\right.$$

After beamforming operation is carried out over the received echo data, the beamformer output can be expressed as

(17) $$\begin{array}{c} \begin{array}{cc} y(f_{l},\tau_{m})= & \frac{{a(f_{l},\theta )}^{H}}{\sqrt{N}}\left(r\left(f_{l},\tau_{m},\theta \right)\right)\\ & =\frac{{a(f_{l},\theta )}^{H}}{\sqrt{N}}(\sqrt{\frac{E}{M}}a(f_{l},\theta )\zeta_{m}\left(f_{l}\right)\beta_{m}\left(f_{l}\right)\frac{G^{*}\left(f_{l}\right)}{\left||G(f_{l})|\right|}\\ & S_{m}^{*}\left(f_{l}\right)e^{-j2\pi f_{l}\tau_{m}}+n_{m}\left(f_{l}\right))\\ & =\sqrt{\frac{EN}{M}}\zeta_{m}\left(f_{l}\right)\beta_{m}\left(f_{l}\right)\frac{G^{*}\left(f_{l}\right)}{\left||G(f_{l})|\right|}S_{m}^{*}\left(f_{l}\right)e^{-j2\pi f_{l}\tau_{m}}\\ & +v_{m}\left(f_{l}\right). \end{array} \end{array}$$

Furthermore, let $h_{m}=\beta_{m}\left(f_{l}\right)\frac{G^{*}\left(f_{l}\right)}{\left||G(f_{l})|\right|}$. Thus, Equation (17) can be rewritten as

(18) $$\begin{array}{c} y\left(f_{l},\tau_{m}\right)=\sqrt{\frac{EN}{M}}h_{m}\left(f_{l}\right)\zeta_{m}\left(f_{l}\right)S_{m}^{*}\left(f_{l}\right)e^{-j2\pi f_{l}\tau_{m}}+v_{m}\left(f_{l}\right). \end{array}$$

For the broadband signal, the beamformer output corresponding to the whole frequency band of the *m*th transmitted signal can be written as

(19) $$\begin{array}{cc} y_{m}= & \sqrt{\frac{NE}{M}}[h_{m}\left(f_{0}\right)\zeta_{m}\left(f_{0}\right)s_{m}\left(f_{0}\right)e^{-j2\pi f_{0}\tau_{m}},\cdots ,\\ & h_{m}\left(f_{L-1}\right)\zeta_{m}\left(f_{L-1}\right)s_{m}\left(f_{L-1}\right)e^{-j2\pi f_{L-1}\tau_{m}}]+v_{m}, \end{array}$$

where $v_{m}=\left[v_{m}\left(f_{0}\right),\cdots ,v_{m}\left(f_{L-1}\right)\right]$. Furthermore, the beamformer output corresponding to all the transmitted waveforms can be expressed as

(20) y=μHζ+v,

where $y={\left[y_{1},\cdots ,y_{M}\right]}^{T}$, $v={\left[v_{1},\cdots ,v_{M}\right]}^{T}$, $\mu =\sqrt{\frac{NE}{M}}$,

(21) $$\begin{array}{cc} H= & diag[s_{1}\left(f_{0}\right)e^{-j2\pi f_{0}\tau_{1}},\cdots ,s_{1}\left(f_{L-1}\right)e^{-j2\pi f_{L-1}\tau_{1}},\\ & \cdots ,s_{M}\left(f_{0}\right)e^{-j2\pi f_{0}\tau_{M}},\cdots ,s_{M}\left(f_{L-1}\right)e^{-j2\pi f_{L-1}\tau_{M}}] \end{array}$$

(22) $$\begin{array}{cc} \zeta = & [h_{1}\left(f_{0}\right)\zeta_{1}\left(f_{0}\right),\cdots ,h_{1}\left(f_{L-1}\right)\zeta_{1}\left(f_{L-1}\right)\cdots ,\\ & h_{M}\left(f_{0}\right)\zeta_{M}\left(f_{0}\right),\cdots ,h_{M}\left(f_{L-1}\right)\zeta_{M}\left(f_{L-1}\right){]}^{T}. \end{array}$$

Due to ζ describing the uncorrelated reflections of different aspects of the target, ζ is assumed to be identical and independent distribution as zero-mean complex random variables with variance 1/ML. In addition, time-reversal transmission results in more acoustic energy on the target and less on other areas for a low level of reverberation, and it is reasonable that v is assumed to be zero-mean complex Gaussian with covariance matrix $\sigma_{n}^{2}I_{ML}$.

Using the matched subspace filtering technique [34], we achieve the test statistic as follows:

(23) $$T=y^{T}P_{H}y=y^{T}I_{ML}y={\left||y|\right|}^{2},$$

where $P_{H}y$ is the components of y that lie in the signal subspace <H>, $I_{MK}$ is a ML×ML unit matrix, and y is given by

(24) $$y=\left\{\begin{array}{c} v,\;H_{0},\\ \mu H\zeta +v,\;H_{1}. \end{array}\right.$$

The distribution of the test statistic *T* in Equation (23) can be expressed as

(25) $$\left\{\begin{array}{cc} T∼\frac{\sigma_{n}^{2}}{2}\chi_{2ML}^{2}, & H_{0},\\ T∼\left(\frac{{\sigma_{n}}^{2}}{2}+\frac{NE}{2M}\right)\chi_{2ML}^{2}, & H_{1}. \end{array}\right.$$

For the narrow-band signal, L=1 is chosen.

Correspondingly, the probability of false alarm can be written as

(26) $$P_{fa}=\mathrm{Pr}\left(\frac{\sigma_{n}^{2}}{2}\chi_{p}^{2}>\gamma \right)=\mathrm{Pr}\left(\chi_{p}^{2}>\frac{2\gamma }{\sigma_{n}^{2}}\right),$$

where p=2M is chosen for the narrow-band signal, and p=2ML for the broadband signal. For a given false alarm rate, the threshold γ is given by

(27) $$\gamma =\frac{\sigma_{n}^{2}}{2}F_{\chi_{p}^{2}}^{-1}\left(1-P_{fa}\right),$$

and the probability of detection can be denoted as

(28) $$\begin{array}{c} P_{d}=\mathrm{Pr}\left(\left(\frac{{\sigma_{n}}^{2}}{2}+\frac{NE}{2M}\right)\chi_{p}^{2}>\gamma \right)=1-F_{\chi_{p}^{2}}\left(\frac{\sigma_{n}^{2}}{\sigma_{n}^{2}\;+\;\frac{NE}{M}}F_{\chi_{p}^{2}}^{-1}\left(1-P_{fa}\right)\right). \end{array}$$

## 3. Numerical Simulations

### 3.1. Exploiting Spatial Diversity for Time-Reversal Focusing

As previously described, time-reversal focusing is utilized in the processing framework for suppressing reverberation and enhancing target echoes. Here, we evaluate the effectiveness of time-reversal transmission by two vertical subarrays with two types of signals. The geoacoustic parameters utilized are similar to those of the at-lake experimental environment as shown in Table 1. The target is assumed to be at the range of 80 m and depth of 10 m. The time-reversal array is also referred to as source-receive array (SRA) in the following content, which consists of eight elements with the inter-element spacing of 2 m. SRA1,SRA2 covers the water column from 1.4 to 15.4 m or from 1.24 to 15.24 m, respectively. A probe source is assumed to be in the vicinity of the target. In addition, 10 ms pulsed continuous wave (PCW) signals at 7 kHz and 9 kHz , linear frequency modulated (LFM) signals at 6–7.5 kHz and 8–9.5 kHz are respectively utilized in numerical simulations. By running the Kraken model [26] with the previous parameters, we can achieve mode functions and wave numbers for analyzing the resolution of time-reversal focusing based on spatial diversity.

**Figure 2:**
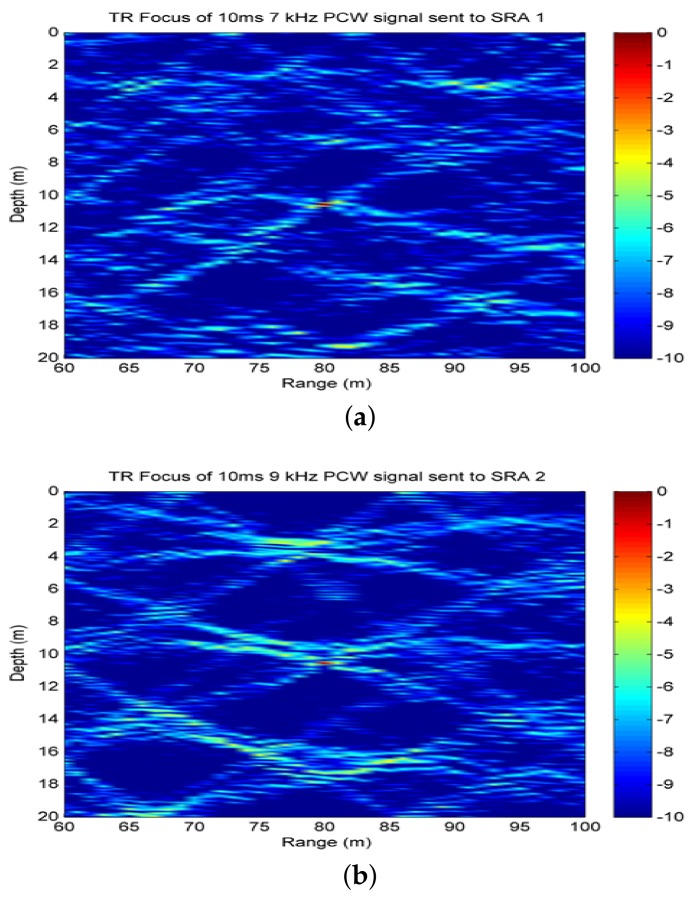
Time-reversal focusing with 10 ms PCW signals: (**a**) 7 kHz signal transmitted by SRA1 covering the water column from 1.4 to 15.4 m ; (**b**) 9 kHz signal transmitted by SRA2 covering the water column from 1.24 to 15.24 m.

**Figure 3:**
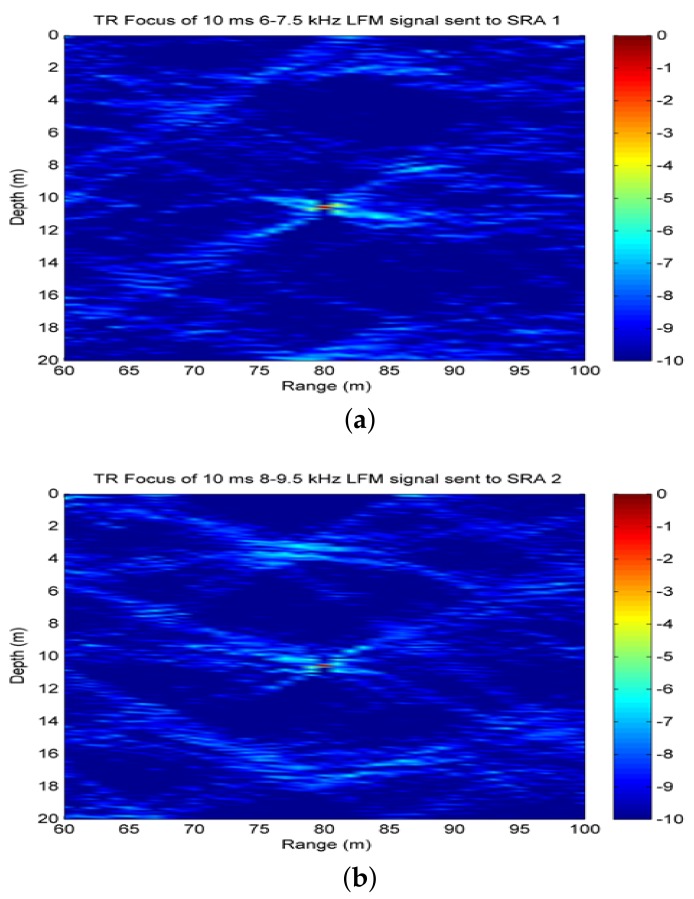
Time-reversal focusing with 10 ms LFM signals: (**a**) 6–7.5 kHz signal transmitted by SRA1; (**b**) 8–9.5 kHz signal transmitted by SRA2.

It is observed from Figure 2 that two focusing spots are both located at (80 m, 10 m) for PCW signals. Moreover, we can see that 9 kHz signal has better resolution than 7 kHz signal by comparing Figure 2b with Figure 2a. For the case, more modes are excited by 9 kHz signal resulting in more interference components. Theoretically, there are the horizontal resolution ΔR=1.17 m and the vertical resolution Δz=0.16 m for 9 kHz while ΔR=1.5 m, Δz=0.2 m for 7 kHz. In addition, the focusing resolution increases with the increase of bandwidth, which is supported by comparing Figure 3 with Figure 2. In Figure 3b, 8–9.5 kHz LFM signal has better resolution due to more modes being excited. The theoretical resolutions for LFM signals are given in Table 2.

### 3.2. Performance Improvement

In this section, detection performance of the distributed TR-MIMO sonar system with the additional vertical diversity gain is evaluated by comparing with that of the distributed phased-MIMO sonar system without the vertical diversity. In the simulation, we consider the similar array configurations for two sonar systems utilized in the at-lake experiments. Two systems have the same receiving array, a 16-element horizontal linear array with the inter-element spacing of 0.075 m. Two vertical arrays are utilized for transmitting time-reversal signals in the distributed TR-MIMO sonar system. SRA1 and SRA2 have the same inter-element spacing of 2 m and cover the water column from 1.4 to 15.4 m or from 1.24 to 15.24 m, respectively. However, in the distributed phased-MIMO sonar system, we consider two horizontal transmitting arrays, each consisting of eight elements with the inter-element spacing of 0.075 m. The target is assumed to be located at the location (80 m, 10 m).

**Figure 4:**
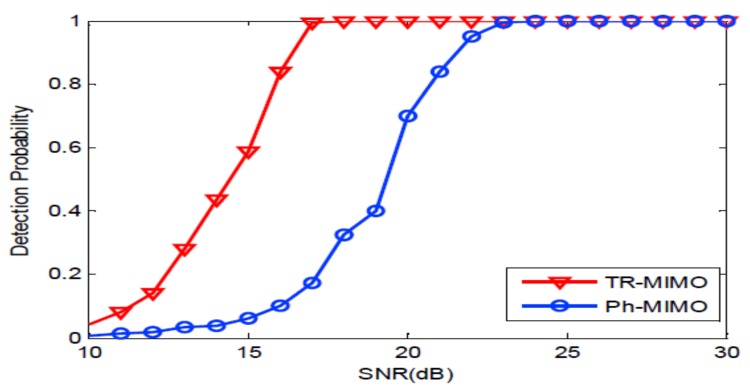
The probability of detection curves as functions of SNR for two sonar systems with a PCW signal. TR-MIMO: distributed TR-MIMO sonar system; Ph-MIMO: distributed phased-MIMO sonar system.

Firstly, we consider the narrow band signals: 10 ms PCW signals at frequency 6 kHz and 8 kHz. Figure 4 depicts the probability of detection curves as functions of SNR for two systems. It can be seen from Figure 4 that SNR of 15.5 dB is required by the distributed TR-MIMO sonar system to achieve the probability of detection ($P_{d}$) of 90% when the probability of false-alarm ($P_{fa}$) of $10^{-3}$ is fixed. Meanwhile, 21 dB is required by the distributed phased-MIMO sonar system to achieve the same detection performance. It is obvious that the TR-MIMO sonar system performs better than the distributed phased-MIMO sonar system due to the former exploiting the vertical diversity. Here, the SNR denoted by β is defined as follows [8]:

(29) $$\beta =\frac{P^{2}N^{2}E^{2}L}{M\sigma_{n}^{4}+PNE\sigma_{n}^{2}+P^{2}N^{2}E^{2}/\left(2M\right)},$$

where L=1 is chosen for the narrow-band signal.

**Figure 5:**
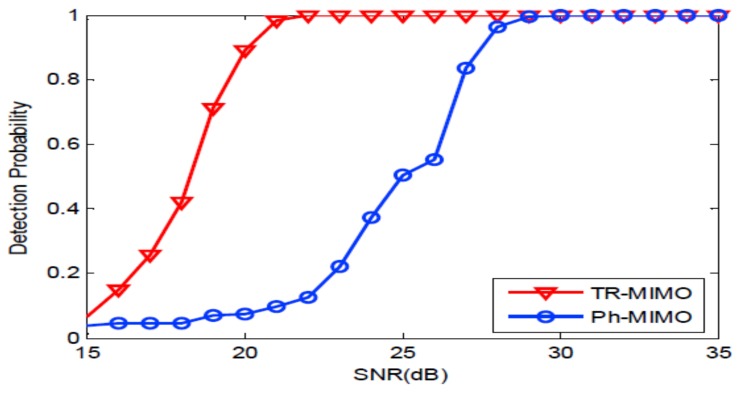
The probability of detection curves as functions of SNR for two sonar systems with LFM signals.

Then, we evaluate the distributed TR-MIMO sonar system with the broadband signals: 10 ms LFM signals with frequency 6–8 kHz and 8–10 kHz. From Figure 5, we can see that the distributed TR-MIMO sonar outperforms the distributed phased-MIMO sonar system due to taking advantage of the vertical diversity for time-reversal focusing. For $P_{fa}=10^{-3},P_{d}=90\%$, the former requires SNR of 21 dB, while the latter requires 27 dB. In addition, it can be seen by comparing Figure 5 with Figure 4 that the distributed TR-MIMO sonar system requires a higher SNR for the FLM signal than for the PCW signal to achieve the same detection performance. For the case, the transmitted power is uniformly distributed over the whole frequency band resulting in a low received SNR.

## 4. At-Lake Experimental Results and Data Analysis

**Figure 6:**
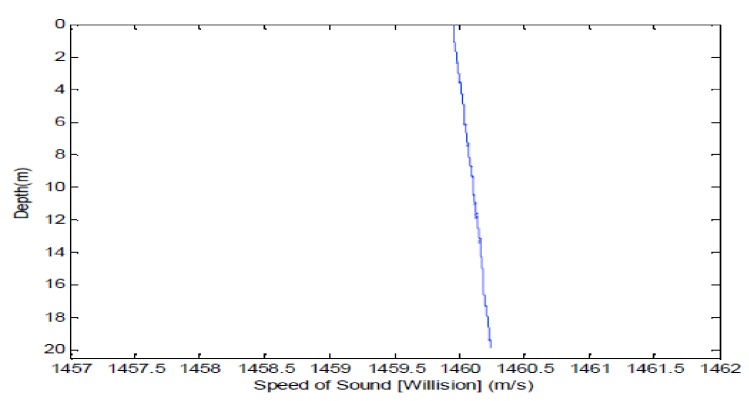
Sound speed profile.

The performance of the distributed TR-MIMO sonar system exploiting spatial diversity is further evaluated by localization of a stationary target at the Moganshan Lake with average water depth of about 20 m. Figure 6 shows sound speed profile measured in the experiment. Clearly, it is a weak positive gradient.

### 4.1. Experimental System

**Figure 7:**
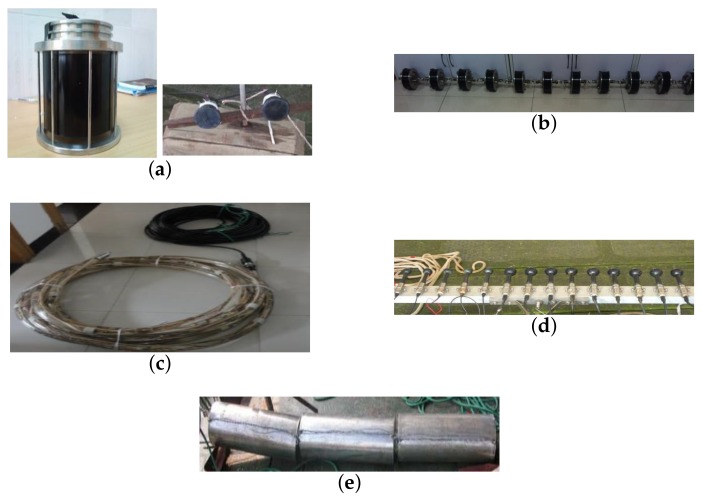
(**a**) probe source (left) and 2-element transmitting array (right); (**b**) 8-element time-reversal array; (**c**) 16-element vertical receiving array for recording time-reversed signals; (**d**) 14-element horizontal array for receiving target echoes; (**e**) target.

Figure 7 shows the distributed TR-MIMO sonar experimental system consisting of a probe source, two 8-element vertical arrays with the inter-spacing of 2 m corresponding to 8 wavelengths at 6 kHz, a 16-element vertical receiving array with the inter-spacing of 1 m, a 14-element horizontal receiving array with the inter-spacing of 0.075 m corresponding to half a wavelength at 10 kHz and a stainless steel cylinder utilized as a target with length of 1.5 m, diameter of 0.2 m and thickness of 0.03 m. Due to the limitation of the experimental conditions, only two 2-element horizontal transmitting arrays are utilized in the distributed phased-MIMO sonar system with the inter-element spacing of 0.093 m as shown in Figure 7a.

### 4.2. Time-Reversal Focusing

**Figure 8:**
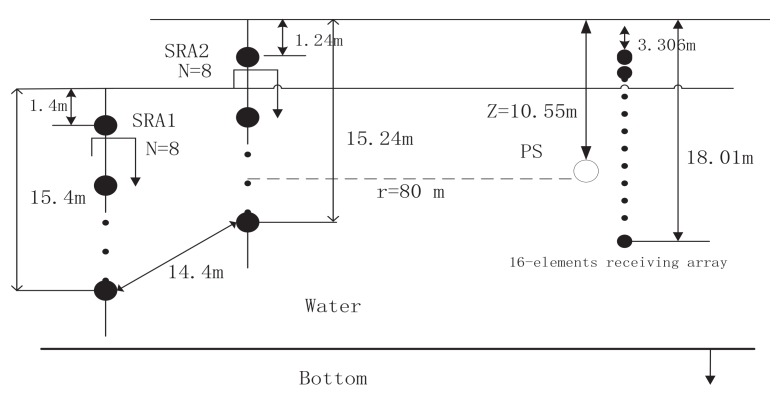
The at-lake experimental configuration for time-reversal focusing. SRA1: time-reversal array1; SRA2: time-reversal array2. SRA1 covers water depth from 1.4 m to 15.4 m. SRA2 covers water depth from 1.24 m to 15.24 m. Distance between SRA1 and SRA2 is 14.4 m. PS: probing source. PS is 80 m far from the center of SRA1 and SRA2; 16-element receiving array covers water depth from 3.306 m to 18.01 m.

Since time-reversal focusing is utilized to enhance target echoes, the first experiment is to observe the spatial-temporal focusing characteristics of the time-reversal PCW signals transmitted by two 8-element vertical arrays. Two time-reversal arrays separated with distance of 14.4 m cover the water column from 1.4 to 15.4 m or from 1.24 to 15.24 m, respectively. The 16-element vertical receiving array is suspended from a target-boat covering the water column from 3.3 to 18.01 m. The range between the target-boat and the time-reversal array is about 80 m. An omni-directional transducer utilized as a probe source is placed at depth of 10.55 m close to the 16-element vertical receiving array. The experimental configuration is shown in Figure 8.

**Figure 9:**
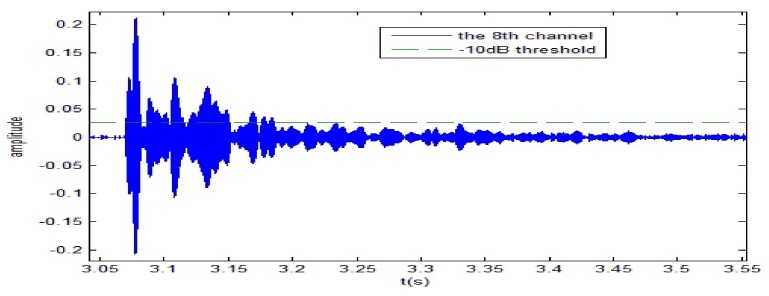
The probing signal at 7 kHz is received by the 8-th channel of SRA1.

**Figure 10:**
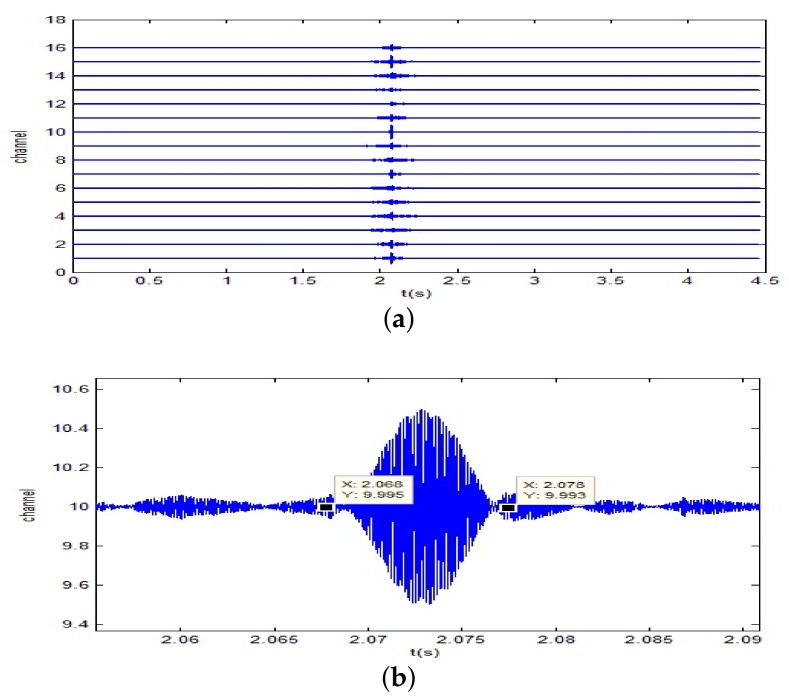
Time reversal-focusing with PCW signal at 7 kHz: (**a**) the time-reversed signals received by the 16-element vertical array; (**b**) local amplification of the signal of the 10th channel.

**Figure 11:**
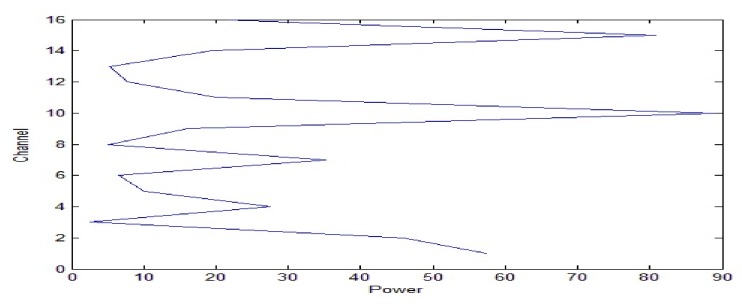
The energy distribution curve of the time-reversed signal at 7 kHz along the depth direction.

**Figure 12:**
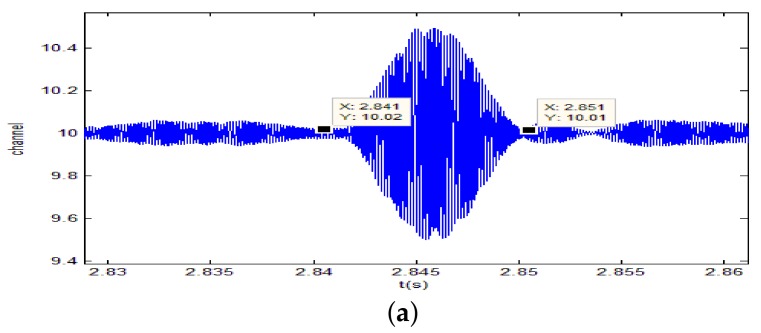

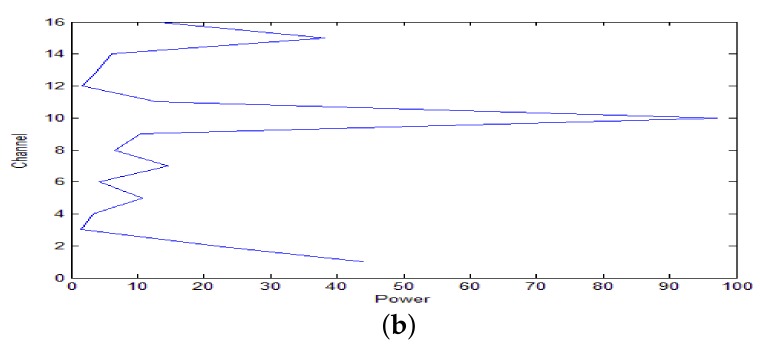
Time-reversal focusing of 9 kHz PCW signal transmitted by SRA2: (**a**) local amplification of the time-reversed signal received by the 10th channel of SRA2; (**b**) the energy distribution curve along the depth direction.

A 10 ms PCW signal at 7 kHz is firstly transmitted by the probing source. Figure 9 shows the signal received by the 8-th channel of SAR1. Due to propagating through the shallow water environment, multipath components result in a time-delay spread of about 100 ms. The dashed line is utilized to decide whether the multipath components exist or not. Here, the threshold is fixed at −10 dB. After energy normalized, SRA1 transmits back the received signal in a time-reversed order. Figure 10a shows the time-reversed signals received by the 16-element vertical array. The local amplification of the signal received by the 10th channel is shown in Figure 10b. It is observed from Figure 10b that the pulse duration of the received time-reversed signal is about 0.01 s. Moreover, the correlation coefficient between the focused signal and the probing signal is 0.994. In addition, one can see from Figure 11 that the highest peak occurs in the 10th channel. Since the site of the 10th element is close to the position of the probe source, it demonstrates that the time-reversed signal can focus on the location of the probe source. The same conclusion can be drawn by SRA2 transmitting the time-reversal PCW signal at 9 kHz as shown in Figure 12.

### 4.3. Target Localization

In the target localization experiment, the 14-element horizonal receiving array is placed between two vertical arrays and located at a depth of 9 m underwater. The target suspended from the target-boat is located at a depth of 10 m. The position of the target is located at ($-8^{\circ }$, 80 m) measured by a global positioning system (GPS). Meanwhile, the 16-element vertical array is moved out of the water. The omni-directional transducer is moved to the front of the 14-element horizonal receiving array for transmitting a probing signal to sense the environment and the likely target. Clearly, with the conditions of $D_{1}+D_{2}=14.4$ m, $\lambda_{\max}=0.25$ m, $R_{0}=80$ m, the array configuration meets the constraint condition Equation (2) to achieve spatial diversity, namely $D_{1}+D_{2}>\lambda_{max}R_{0}/1.5=13.3$ m. For the sake of comparison, two transmitting arrays of the distributed phased-MIMO sonar system are also placed at two ends of the 14-element receiving array with a distance of 13.35 m and depth of 9 m. In the experiments, the constant transmitted power is fixed for two sonar systems.

**Figure 13:**
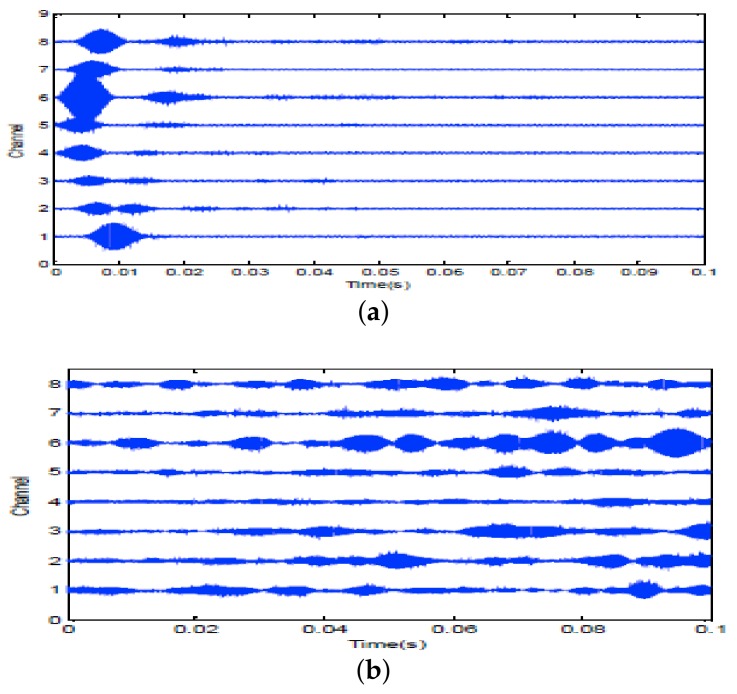
7 kHz PCW signal: (**a**) echoes received by SRA1 after transmitting a probing signal; (**b**) the time-reversed signal for second transmission.

**Figure 14:**
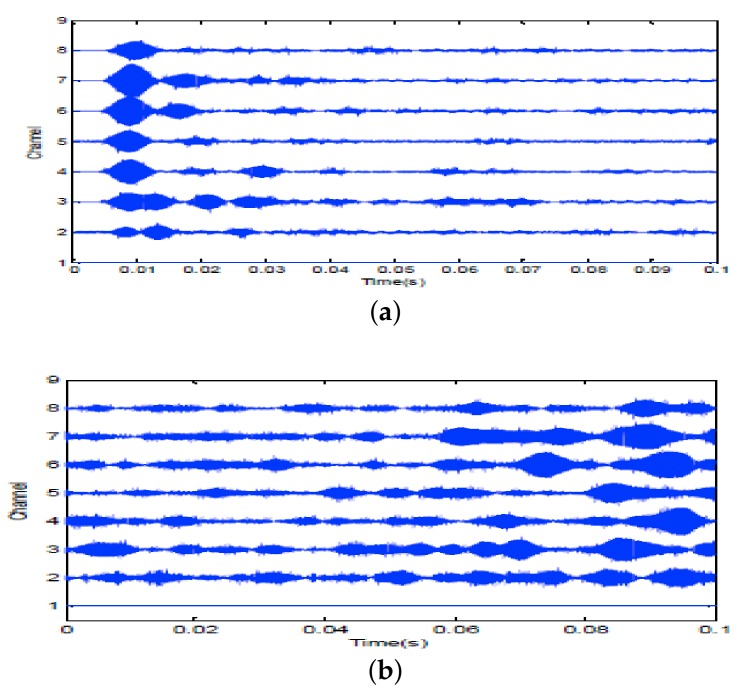
9 kHz PCW signal: (**a**) echoes received by SRA2 after transmitting a probing signal; (**b**) the time-reversed signal for second transmission.

**Figure 15:**
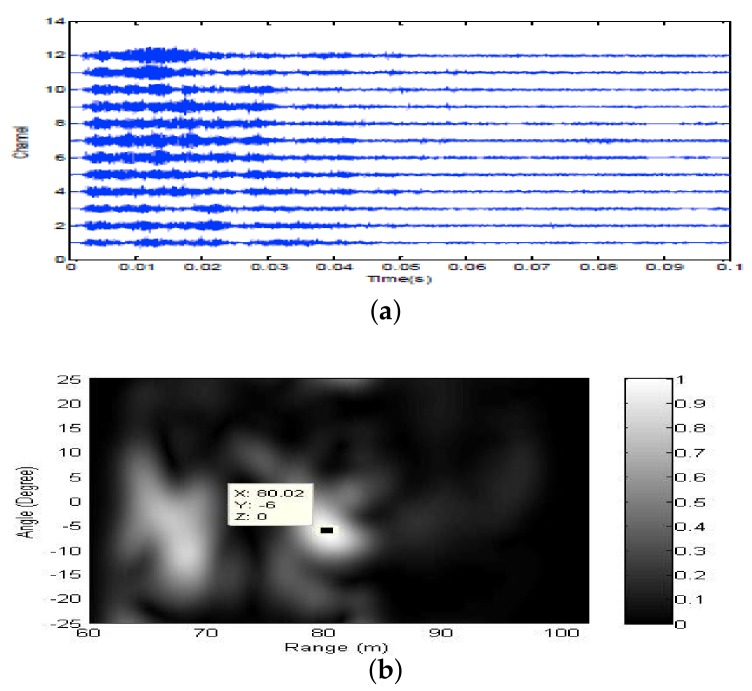
PCW signals: (**a**) the enhanced echoes received by the 14-element horizontal array; (**b**) the ambiguity surface of the output of the distributed TR-MIMO sonar system.

**Figure 16:**
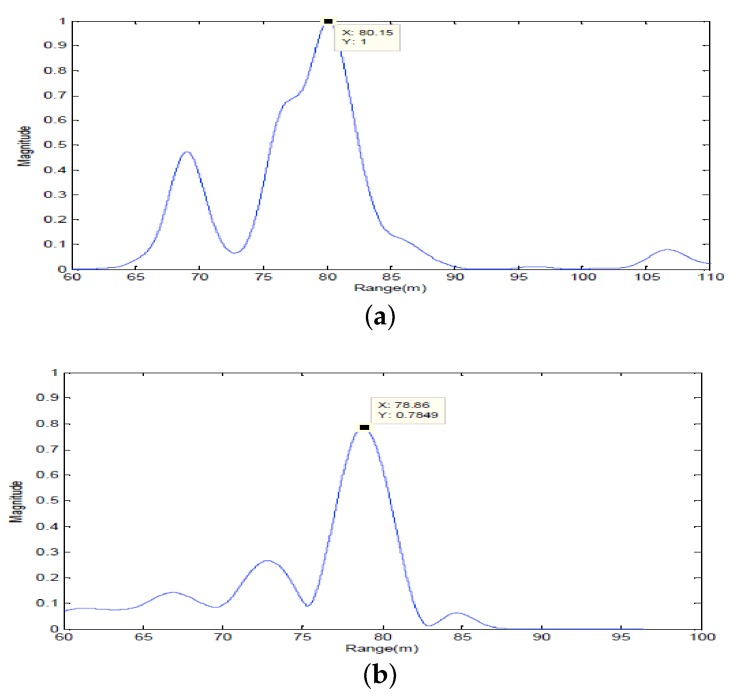
The outputs of the replica correlation integration processer with PCW signals: (**a**) the distributed TR-MIMO sonar system; (**b**) the distributed phased-MIMO sonar system. Note that the outputs are normalized by the maximum value of outputs of the distributed TR-MIMO sonar system.

Firstly, two 10 ms PCW signals at 7 kHz and 9 kHz are respectively transmitted by an omnidirectional transducer and the corresponding echoes are respectively received by SRA1 and SRA2 as shown in Figure 13a and Figure 14a. After amplitude normalization, the received signals are respectively transmitted back in a time-reversed order by two SRAs as shown in Figure 13b and Figure 14b. In experiments, a transducer (the 1-st channel) of the SAR2 is not operating correctly and disabled. When the target is simultaneously illuminated by two time-reversed signals, the enhanced target echoes are recorded by the 14-element receiving array as shown in Figure 15a. From the ambiguity surface in Figure 15b, we can see that the target is located at ($-6^{\circ }$, 80.02 m), which is close to the true target position. Figure 16 shows the range estimates of two sonar systems, respectively. It is obvious that the distributed TR-MIMO sonar system has a high correlation peak due to the vertical diversity gain. The received SNR is 20.61 dB in the former while 19.02 dB in the latter. Note that all outputs are normalized by the maximum value of the replica correlation integration processer outputs of the distributed TR-MIMO sonar system.

**Figure 17:**
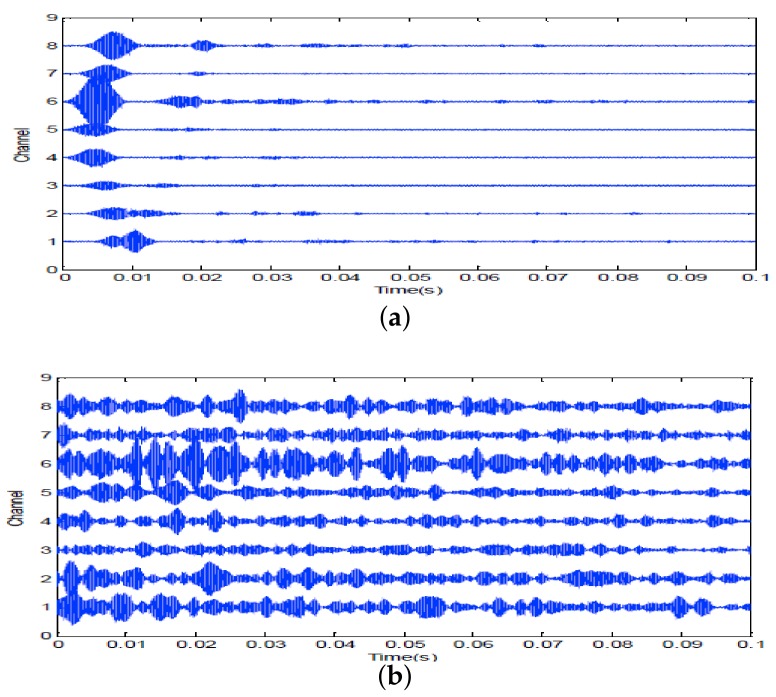
LFM signal at 6–7.5 kHz: (**a**) echoes received by SRA1 after transmitting a probing signal; (**b**) the time-reversed signal for the second transmission.

**Figure 18:**
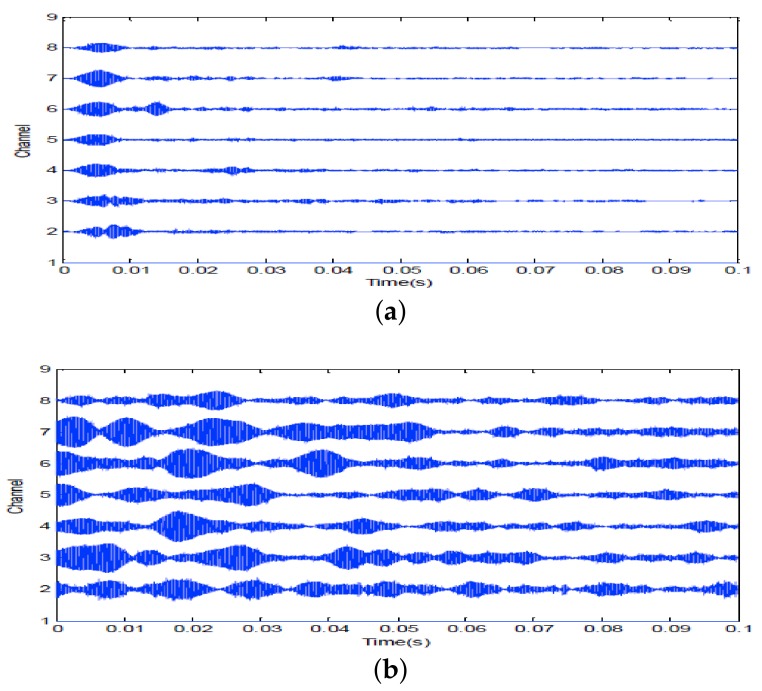
LFM signal at 8–9.5 kHz: (**a**) echoes received by SRA2 after transmitting a probing signal; (**b**) the time-reversed signal for second transmission.

**Figure 19:**
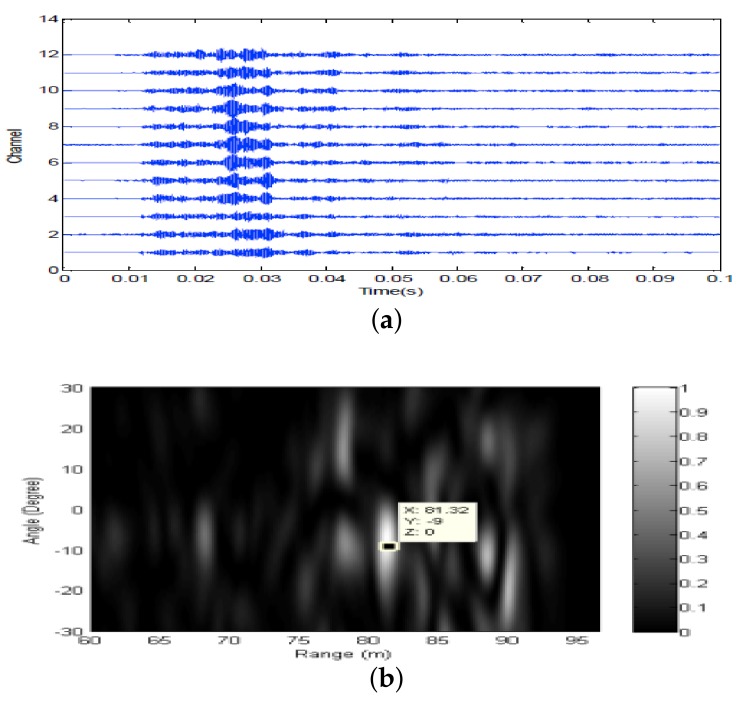
LFM signals: (**a**) the enhanced echoes received by the 14-element horizontal array; (**b**) the ambiguity surface of the output of the distributed TR-MIMO sonar system.

**Figure 20:**
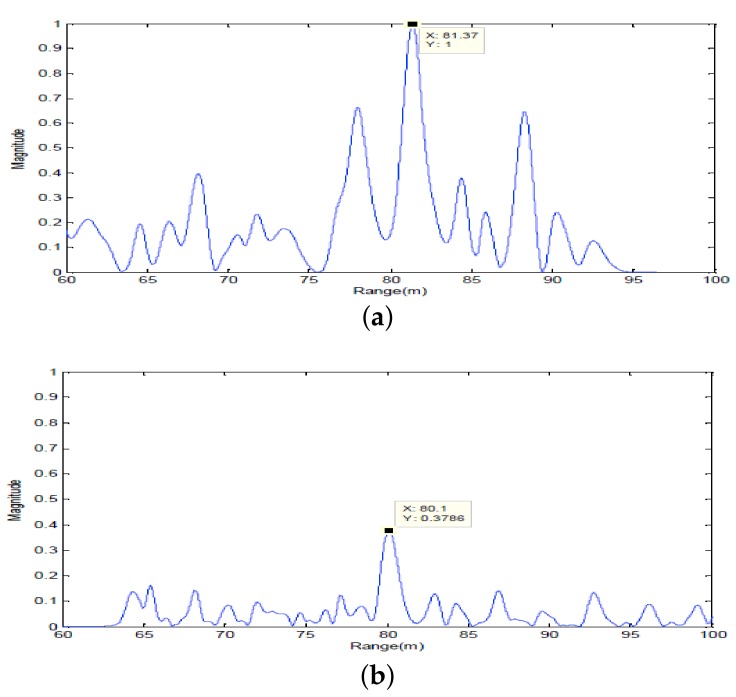
The outputs of the replica correlation integration processer with LFM signals: (**a**) the distributed TR-MIMO sonar system; (**b**) the distributed phased-MIMO sonar system. Note that the outputs are normalized by the maximum value of the outputs of the distributed TR-MIMO sonar system.

Then, the omnidirectional transducer respectively transmits 10 ms LFM signals at 6–7.5 kHz and 8–9.5 kHz. Figure 17a and Figure 18a respectively show the echoes received by two SRAs. When the target is simultaneously illuminated by two time-reversed LFM signals (Figure 17b and Figure 18b), the enhanced target echoes are shown in Figure 19a. Figure 19b shows the corresponding ambiguity surface. From Figure 19b, we can see that the target is located ($-9^{\circ }$, 81.32 m) close to the true target position. Figure 20 shows the outputs of the replica correlation integration processer of two sonar systems, respectively. Clearly, the distributed TR-MIMO sonar system performs better than the distributed phased-MIMO sonar system due to exploiting the vertical diversity. The former has the received SNR 21.68 dB, and the latter has 17.02 dB. In addition, it is interesting to see that a high resolution in range is achieved by the broadband LFM signal by comparing Figure 20 with Figure 16.

## 5. Conclusions

In this paper, we have proposed a coherent and noncoherent jointly sonar processing framework to combine spatial diversity gain with beamforming array gain for active detection. Due to the exploiting of spatial diversity for time-reversal focusing resulting in a low level of reverberation, the sonar experimental system works well in shallow water, which has been verified by localization of a small target in the at-lake experiment. The robustness of time-reversal processing comes from the iterative strategy, transmitting a PS to sense environments and echo of a likely target for guiding the second transmission. By illuminating the target from multiple angles, it is possible for the distributed sonar system to solve the problem of detection performance degradation due to target scintillation.

## Figures and Tables

**Table 1:** Geoacoustic parameters.

Type	Thickness (m)	Density (g/cm${}^{3}$)	Speed (m/s)	Absorption (dB/λ)
Water	20	1.0	1460	0.0
Sediment	5	1.8	1700	0.5
Bottom	∞	3.0	3000	2.0

**Table 2:** Resolution of time-reversal focusing.

Frequency (kHz)	Δ R (m)	Δ z (m)
7	1.5	0.2
9	1.17	0.16
6–7.5	1.54	0.21
8–9.5	1.12	0.16
